# Effectiveness of MTA apical plug in dens evaginatus with open apices

**DOI:** 10.1186/s12903-021-01920-6

**Published:** 2021-11-08

**Authors:** Khoa Van Pham, Thu Anh Tran

**Affiliations:** 1 Department of Operative Dentistry and Endodontics, Faculty of Odonto-Stomatology, University of Medicine and Pharmacy at Ho Chi Minh City, Ho Chi Minh City, 700000 Vietnam; 2 Department of Operative Dentistry and Endodontics, National Hospital of Odonto-Stomatology, Ho Chi Minh City, Vietnam

**Keywords:** MTA, Root canal treatment, Open apex, Apical plug, Collagen

## Abstract

**Background:**

The present study aims to evaluate the effectiveness of mineral trioxide aggregate (MTA) application in treating dens evaginatus affected teeth with apical lesions and open apices using haemostatic collagen membrane to prevent the apical extrusion of MTA.

**Methods:**

Twelve patients with 14 dens evaginatus affected teeth with apical lesions and open apices were treated with MTA apical plug and haemostatic collagen membrane. Clinical symptoms of subjective pain, pain of palpation, percussion, sinus tract, and the apical lesions' radiographic parameter were recorded at every 3-month interval up to 9 months after treatment. Paired t-test or Wilcoxon signed-rank test was used for statistical analysis with P < 0.05 as the threshold for considering results to be statistically significant.

**Results:**

No patient experienced clinical symptoms 3 months after endodontic treatment. In addition, there was a significant difference in the dimensions of the apical lesions' before compared to 3 months after endodontic treatment.

**Conclusions:**

The combination of MTA apical plug and haemostatic collagen membrane effectively treated dens evaginatus affected teeth with apical lesions, and open apices.

## Background

Dens evaginatus is an uncommon anomaly on the occlusal central groove or lingual ridge of the buccal cusp of the posterior tooth [1]. These developmental aberrations or occlusal supernumerary cusps have many description terminologies, such as tubercle, excrescence, protuberance, and bulge; they project on the neighbouring enamel structure, including the dentinal core and pulp horn as well [2]. With a high occurrence rate (0.5–4.3%) in Asians [3], this abnormal cusp has become a special feature of the Asian populations and gets critical clinical significance versus the Carabelli cusp without pulp in the Caucasian population [2]. Pulp vitality conservative procedures for this unnatural entity include gradually decreasing the cusp height and all opposing occlusal interferences or composite build-up surrounding the cusp until the pulpal recession occurs [1]. Because of the considerable height of the dens evaginatus tubercle on the occlusal surface, malocclusion is inevitable in these cases. Caries has not been considered as the main aetiology of the pulpal diseases for this entity, but occlusal interferences are suggested as the main reason resulting in pulp exposure because of a broken-down cusp or worn dentin layer [2], periapical periodontitis, and further changes in periodontal connective tissue [4]. The most critical issue is the early recognition and well-timed management of the situation whenever the affected tooth erupts into the position to prevent further pathology [2]. In contrast to the tooth with a mature apex, which can be treated with the normal procedure without special requirements, a tooth with an immature apex or open apex, especially with a periapical component, can only be managed with time-consuming, difficult, distinctive, and complicated therapy to conserve the tooth. One of the most historical and longest procedures for this situation is apexification with Ca(OH)_2_ paste placement into the canal to induce the formation of a calcific barrier at the apex of the root [5]. Along with the drawback of prolonged therapy, apexification with Ca(OH)_2_ induces weakness of the root and tooth structure with a reduction in fracture strength, i.e. by about one half after one year of investigation [6]. Chemical or thermal gutta-percha techniques for apical third obturation have proved ineffective; moreover, these are sensitive techniques and require certain difficult conditions [7]. With the advancements of material technology, introducing calcium silicate-based cement promises a better clinical outcome for the open apex situation with a brief clinical procedure [7]. Possessing good biological and physicochemical properties, mineral trioxide aggregate (MTA) is advocated as a promising material for this complicated procedure although it presents some shortcomings such as a long setting time, specific moisture in the environment during setting, and the potential inflammatory induction if accidentally pushed into the apical region [7]. In order to prevent the apical extrusion of MTA, an additional apical membrane with collagen has been advocated, prior to the insertion of MTA into the apical third of the root canal [8, 9]. However, high expense and difficult manipulation are two major drawbacks in the application collagen membranes for the apical extrusion of MTA. A valuable alternative to the collagen membrane is a collagen sponge with low cost and easy manipulation [7]. The present study aims to evaluate the effectiveness of MTA application in treating dens evaginatus affected teeth with apical lesions and open apices using haemostatic collagen membrane to prevent the apical extrusion of MTA.

## Materials and methods

This study was approved by the Research Ethics Committee of the University of Medicine and Pharmacy at Ho Chi Minh City, Viet Nam, with the approval number of 195/ĐHYD-HĐĐĐ. Informed consent was obtained from parents of all participants, or participant herself, and all methods were performed in accordance with the relevant guidelines and regulations. Informed consent was also obtained from all subjects and/or their legal guardian(s) for the publication of identifying information/images in an online open-access publication. The sample size calculation was performed using G*Power version 3.1.9.6 (Universität Kiel, Germany) with the effect size of 0.85, alpha = 0.1, and power of 0.9, resulting in a sample size of 14 teeth. The Wilcoxon signed-rank test (matched pairs) in the t-test family was used for the reduction of the periapical lesion dimension before and after endodontic therapy. Mandibular premolars with dens evaginatus were selected for the present study with one root canal tooth, diagnostic of symptomatic or asymptomatic apical periodontitis, periapical lesion, and open apex. The exclusion criteria included orofacial chronic pain such as migraine, temporomandibular pain, sinusitis, and trigeminal neuralgia. Teeth that were unrestorable, with an unfavourable crown-to-root ratio, or cracked were also excluded from the study. The recruitment of patients was performed at the Department of Operative Dentistry and Endodontics, National Hospital of Odonto-Stomatology, Ho Chi Minh City, Viet Nam, from May 2019 to December 2020.

**Fig. 1:**
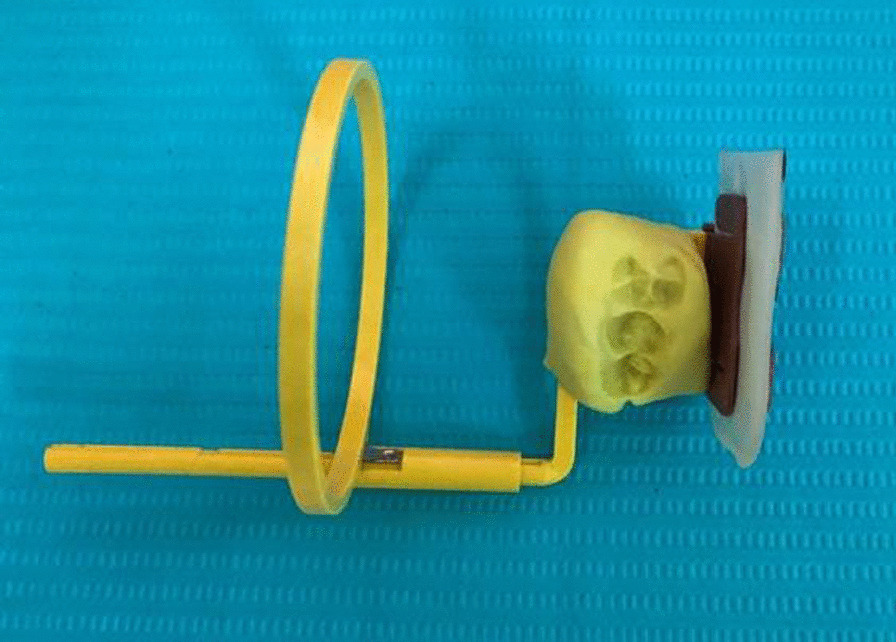
Individual bite registration impression in the 16-inch position device

**Fig. 2:**
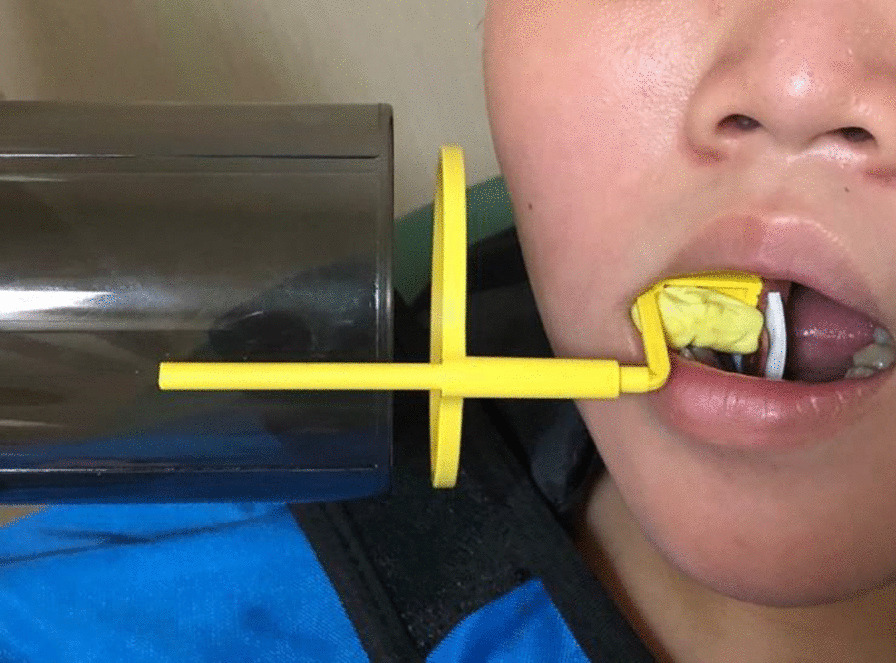
Bite registration in place on the clinical setting

**Fig. 3:**
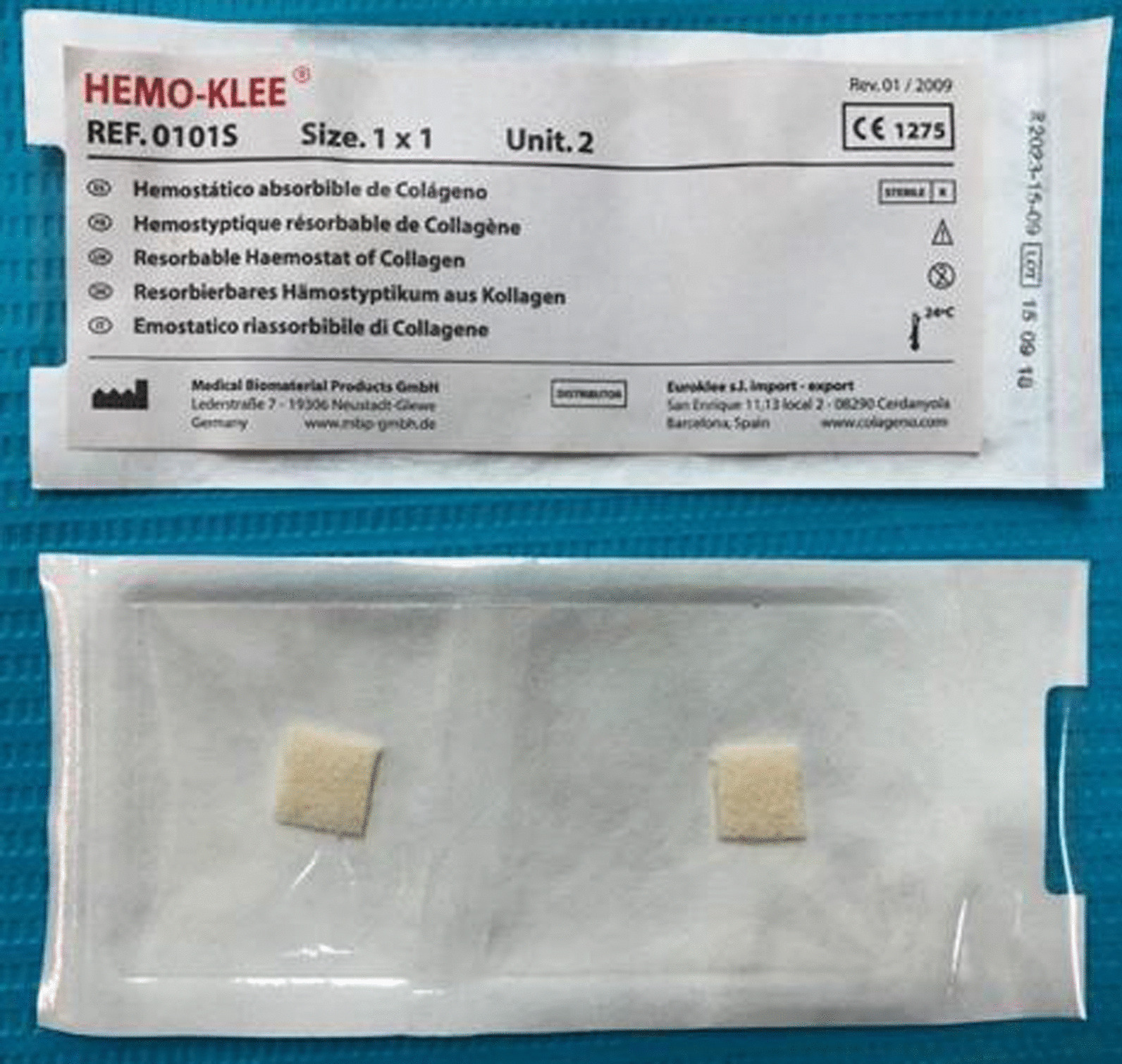
Hemostatic collagen Hemo-Klee

**Fig. 4:**
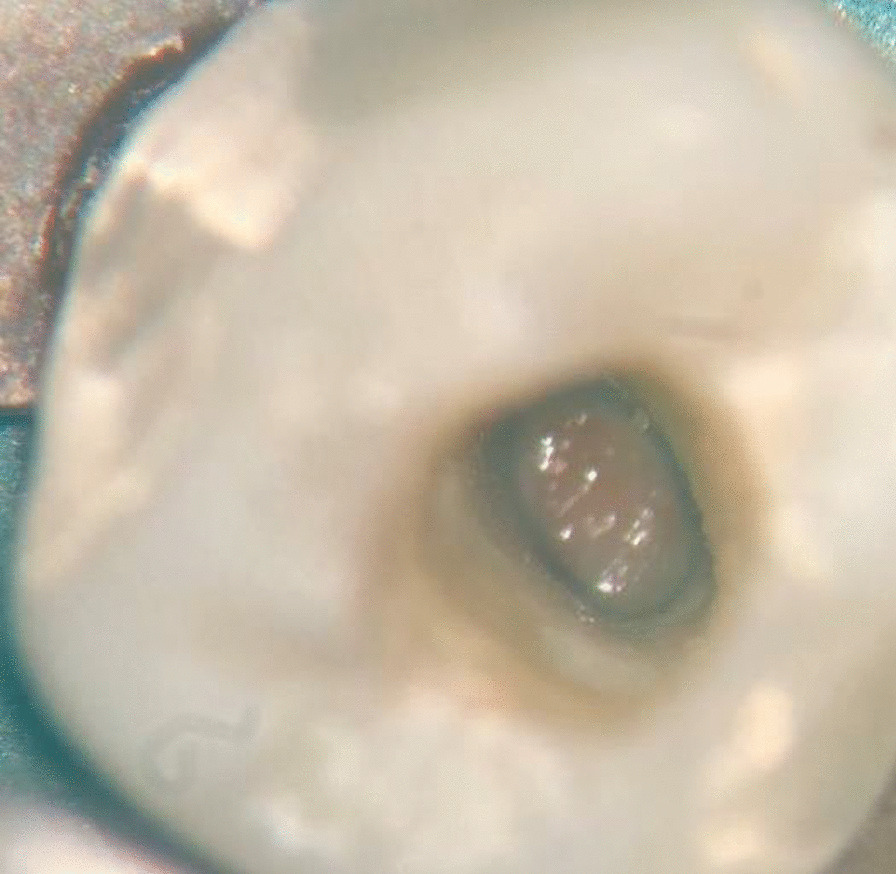
Collagen sponge in placement at the apical region of the root canal

**Fig. 5:**
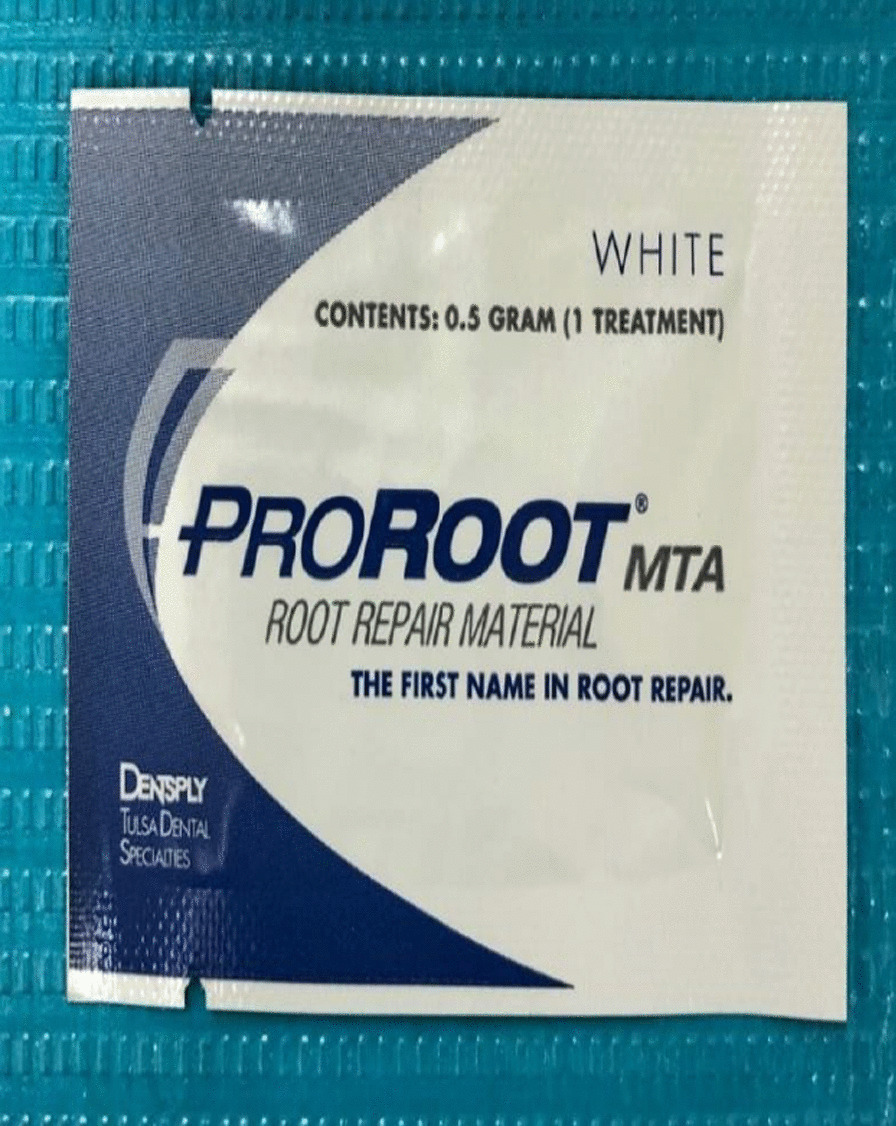
ProRoot MTA (Dental Tulsa Specialties, Tulsa, Oklahoma, USA)

**Fig. 6:**
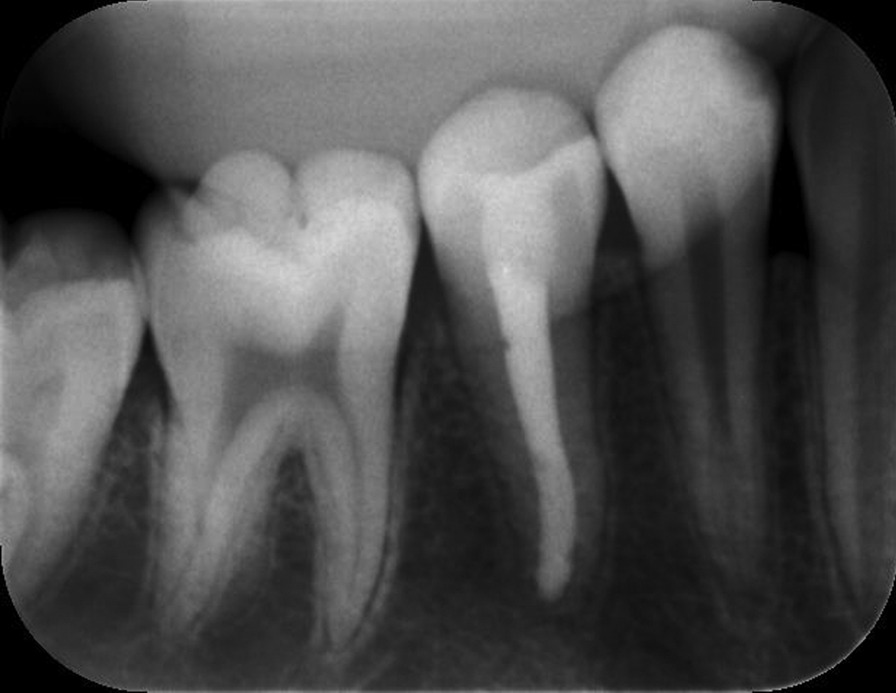
Radiography of the second premolar after 3 months of treatment

**Fig. 7:**
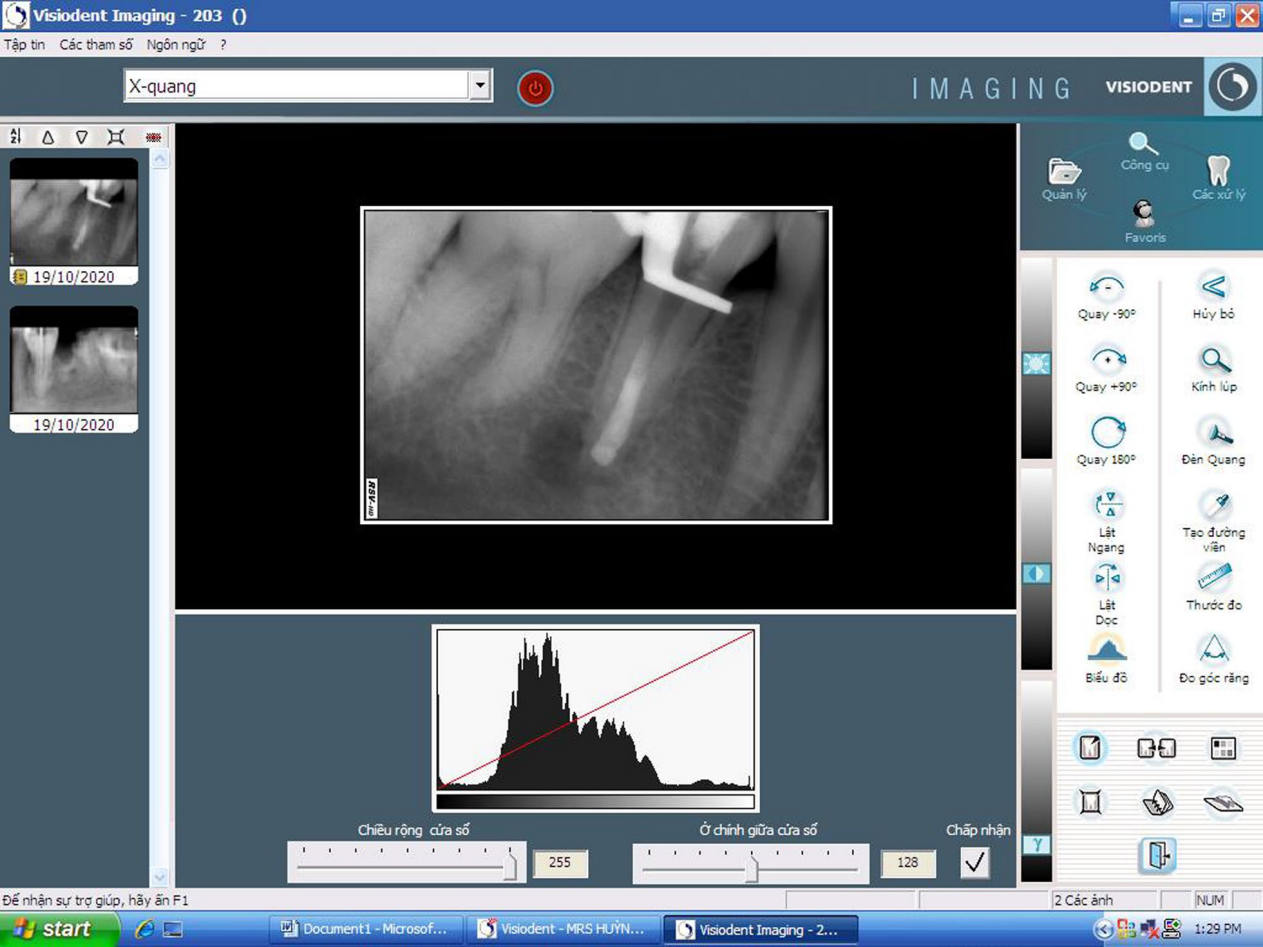
Visiodent software interface for measurements of apical lesion dimension

**Fig. 8:**
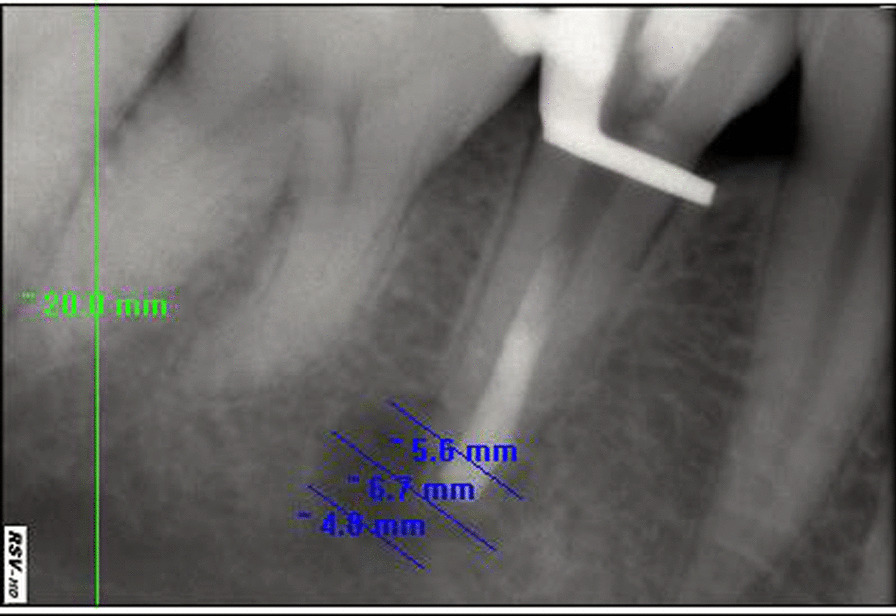
Measurement of apical lesion dimension at closer look

All root canal procedures were performed by the same endodontist with the same standard procedure. For the first appointment, after the administrative procedure was completed, a silicone bite registration impression was established to expose the first digital long cone periapical radiograph using the X-Mind unit and the phosphor plate (Satelec, Acteon Group, France) with a 16-inch position device (Figs. 1 and 2). Local anaesthesia was delivered using 2% lidocaine (Lignospan standard, Septodont, France), following by rubber dam isolation (Ash Rubber Dam, Dentsply Sirona, Switzerland). After this stage, a dental operating microscope (Opmi Proergo, Zeiss, Germany) was used for the entire endodontic procedure. An access cavity was prepared using an Endo-Z and Martin bur (Dentsply Sirona, Maillefer, Switzerland), with copious sterile water spray. The working length then was determined by a combination of the electronic apex locator (ProPex II, Dentsply Sirona, Maillefer, Switzerland) and the periapical digital radiograph. The root canal was prepared to 2 mm short of the root canal length using K-files (Dentsply Sirona, Maillefer, Switzerland) with gentle, circumferential instrumentation, and copious irrigation with 3% sodium hypochlorite (Canal Pro, Coltene Whaledent, Altstätten, Switzerland) using a Max-i-Probe irrigation needle (Dentsply Sirona, Maillefer, Switzerland). The endodontic preparation was performed with the aim of maximum content cleaning and minimal dentin removal. The root canal was finally irrigated using sterile saline solution and dried with sterile paper points. Calcium hydroxide paste (Endo Cal, Septodont, France) was extruded from the syringe into the root canal and gently condensed using an appropriate plugger (Dentsply Sirona, Maillefer, Switzerland), ensuring the paste was filled up from the apical end to the cemento-enamel junction. The access cavity was then temporarily filled using Cavit (GC, Tokyo, Japan) with an underlying cotton pellet. The second appointment was set for 7 days after the first one. The canal content was cleaned with a file and sodium hypochlorite irrigation after removal of the temporary filling, dried with sterile paper points, and prepared for the apical plug procedure. Resorbable haemostatic collagen (Hemo-Klee, Medical Biomaterial Products GmbH, Germany) was adjusted, inserted, and gently condensed into the apical apex region with an appropriate plugger (Fig. 3). Under the microscope, the pieces of cut collagen membrane were condensed incrementally through the canal into the periapical area using the proper condenser until a firm barrier was established at the apex of the root. The procedure was performed rapidly and easily because of the widening of the apical portion of the root. The position and the texture of the collagen barrier were checked for stability with the plugger under the microscope and additional layers of collagen could be further condensed into place if needed (Fig. 4). White MTA (ProRoot MTA, Dentsply Sirona, Tulsa Dental Specialties, Oklahoma, USA) was prepared according to the manufacturer’s instructions and placed in the apical region, reaching a depth of 5 mm using the MTA carrier and appropriate plugger (Fig. 5). A digital radiograph was captured to ensure that the procedure had been performed appropriately. With a moistened cotton pellet on the top of the MTA apical plug, and another dry one for the remainder of the canal, the access cavity was temporarily filled with Cavit for another four days before the third appointment. At this appointment, the hardness of the coronal MTA surface was probed with a plugger after removal of the temporary Cavit and cotton pellets. The remaining canal space was obturated with thermoplastic gutta-percha (EQV, Meta Biomed, Korea) and AH Plus sealer (Dentsply Sirona, Maillefer, Switzerland), and the access cavity was filled up with composite (GC, Tokyo, Japan) and a liner of glass ionomer (GC, Tokyo, Japan) after ensuring the obturation quality with a digital radiograph. Periodical clinical and radiographical examinations (using the individual bite registration impression ensuring the same position of tooth before and after procedure) were performed with 3-month intervals for the follow-up period of 9 months (Fig. 6). Clinical symptoms included postoperative pain and analgesic consumption, pain on palpation or percussion, and sinus tract. Clinical failure was recorded whenever there were any of the above clinical symptoms. Radiographic parameters provided the periapical lesion dimension. This dimension was determined as the largest width of the segment from the two intersections between the lesion circumferential and line drawn perpendicular to the root axis (Figs. 7 and 8). Radiographic success was established whenever there was a reduction in the periapical lesion dimension. The continued formation of the root apex was also recorded. All evaluations were performed by the same endodontist with the Kappa (for clinical findings), or Intra-Class Correlation (for radiographic parameters) coefficients used to assess intra-examiner agreement. Statistical analysis was performed using MedCalc Statistical Software version 19 (MedCalc Software, Ostend, Belgium) with a significance level of p < 0.05. Data were first tested for normality by the Shapiro–Wilk test to use the paired t-test. If the data were not normally distributed, the data were analysed using the Wilcoxon signed-rank test. The correlations among the variables such as age, sex, lesion dimensions at different times, and continued root development were performed using the Pearson correlation test.

## Results

There were 12 patients included in the study. The youngest patient was 9-years-old, and the oldest patient was 22-years-old with seven males and five females. There was one patient with three teeth with dens evaginatus in the present study. All teeth in the present study were lower premolars.

The Kappa value was higher than 0.96 and the ICC indices were greater than 0.9, indicating that the intra-examiner agreement was high.

The results of clinical findings of the subjects are displayed in Table 1. There were no clinical symptoms in the subjects at all experimental intervals. All symptoms ceased completely after 3 months of treatment.

**Table 1:** Periodical clinical findings of the subjects

Clinical findings	3 months	6 months	9 months
Postoperative pain	0	0	0
Pain of palpation	0	0	0
Pain of percussion	0	0	0
Sinus tract	0	0	0

The periapical lesion dimensions and its changes are displayed in Table 2. There was a statistically significant reduction in the dimension at even the first 3-month interval. The mean reduction was approximately 3.5 mm after 9 months.

**Table 2:** Periodical radiographical parameter of the subjects (Mean ± Standard Deviation – mm)

Radiographic parameters	Before treatment	3 months	6 months	9 months
Dimension	3.89 ± 0.97	2.06 ± 1.06	0.93 ± 1.21	0.42 ± 0.74
Mean of reduction	N/A	1.82	3.01	3.50
P values	< 0.0001*			
	0.0001^†^			
	0.0001^†^			

^*,†^P < 0.05: significantly statistical differences

*Paired t-test

^†^Wilcoxon signed rank test

The Pearson correlation coefficients among age, sex, and lesion dimensions at different examined time points (before treatment, then at 1, 2, 3, 6, and 9 months after treatment) are displayed in Table 3. There were significant correlations between the lesion dimensions before treatment and at 1, 2, and 3 months after treatment. There were also significant correlations between the lesion dimensions at 3, 6, and 9 months after treatment.

**Table 3:** The Pearson correlations among variables

		Age	Sex	T0	T1	T2	T3	T6	T9	CDR
Age	PC	1	.326	− .114	− .069	.000	.091	.118	.107	− .110
	Sig		.255	.698	.814	1.000	.757	.688	.715	.709
Sex	PC	.326	1	− .445	− .435	− .321	− .157	− .007	− .072	.174
	Sig	.255		.111	.120	.263	.592	.982	.807	.552
T0	PC	− .114	− .445	1	.973*	.801*	.708*	.429	.430	− .427
	Sig	.698	.111		.000	.001	.005	.126	.125	.128
T1	PC	− .069	− .435	.973*	1	.817*	.757*	.465	.437	− .490
	Sig	.814	.120	.000		.000	.002	.094	.119	.075
T2	PC	.000	− .321	.801*	.817*	1	.860*	.719*	.663*	− .190
	Sig	1.000	.263	.001	.000		.000	.004	.010	.514
T3	PC	.091	− .157	.708*	.757*	.860*	1	.898*	.766*	− .490
	Sig	.757	.592	.005	.002	.000		.000	.001	.075
T6	PC	.118	− .007	.429	.465	.719*	.898*	1	.895*	− .417
	Sig	.688	.982	.126	.094	.004	.000		.000	.138
T9	PC	.107	− .072	.430	.437	.663*	.766*	.895*	1	− .303
	Sig	.715	.807	.125	.119	.010	.001	.000		.292
CDR	PC	− .110	.174	− .427	− .490	− .190	− .490	− .417	− .303	1
	Sig	.709	.552	.128	.075	.514	.075	.138	.292	

*PC* Pearson Correlation, *Sig.* Significant 2-tailed, *CDR* Continue Development Root, *T0* Lesion dimension before treatment, *T1* Lesion dimension after 1 month, *T2* Lesion dimension after 2 months, *T3* Lesion dimension after 3 months, *T6* Lesion dimension after 6 months, *T9* Lesion dimension after 9 months

*Correlation is significant at the 0.01 level (2-tailed)

## Discussion

The results of the present study show that there were no clinical symptoms 3 months after endodontic treatment in all cases. The apical lesion dimensions were significantly reduced in the second month, after apical plug creation with MTA treatment. There were high correlations among periapical lesion dimensions at different time points. The results reveal that the lesion dimensions reduced quickly each month after the second month at a high rate.

The pain symptoms disappeared 3 months after endodontic therapy in the present study. This result agrees with that of previous studies [8, 10–13]. The results of the present study differ from previous studies [14, 15] in that there were one or two cases with pain symptoms after the last appointment. Swelling had ceased completely after the last appointment in the present study, in agreement with others [10–13]. However, this result disagrees with that of a German study [14].

The clinical examination result revealed that, in the first month after therapy, over 95% of cases could be confirmed as successful without any clinical symptoms, and 3 months after treatment, the success rate was 100% in the present study. This result agrees with that of previous studies [10, 12] with the same 100% successful clinical outcome. The results of the present study are better than those of longer studies [16, 17], with one and two years of follow-up, respectively.

The results of apical lesion healing on the periapical digital radiograph in the present study agrees with those of a previous study [14]. The apical lesion reduction level of 3.5 mm after 9 months of follow-up in the present study was a little lower than that the 4 mm observed after 33 months of follow-up in a previous study [14]. At the 6-month follow-up appointment, nearly half of cases were completely healed and at the end of the 9-month follow-up period, two thirds of cases were completely healed. These results are similar to those of previous studies [13, 18]. The present results are better than those of previous study [8] but not as good as another study [15], although the present study did disclose the before-treatment apical lesion dimensions. There was no increase in the apical lesion dimension in all cases in the present study, which agrees with the results of a previous study [8], and differs from others [18, 19]. The difference among the studies might be due to the utilisation of biocompatible collagen as a barrier before the insertion of MTA into the apical region; in this study, we used resorbable haemostatic collagen. The collagen barrier used as a membrane prevented the extrusion of MTA into the periapical region, which could irritate the periapical tissue [20, 21]. Collagen membranes are commonly used in periodontology and recently, with apical plug creation, have provided promising results in the healing of periapical lesions [7].

MTA apexification does not contribute to fracture resistance despite saving time in the procedure [22]. A non-vital immature tooth becomes more susceptible to fracture because of the thin dentinal walls [23, 24]. MTA apexification is an alternative to the regenerative endodontic therapy option [11]. The stage of root development is a very important factor in the restorable capability and the survival of the tooth, especially with an apexification procedure [24, 25].

The bite registration index was introduced in a previous study for one or multiple visits in endodontics [26]. Along with the measurement of the largest diameter of the periapical lesion, the bite registration index helps to search for the correct previous position of the sensor and the long cone of the X-ray machine for a reliable measurement of the dimensions for comparison of the results after periodic intervals. This manipulation was simple, easy, and fast in the clinical setting and can be used for further investigations in endodontic therapy. Regarding the measurement of the periapical lesion dimension, one study used the technique of direct measurement on the magnified periapical radiograph using an eyepiece graticule [8], while another did not provided detailed descriptions [14]. The present study used the integrated software from the manufacturer to measure all dimensions with reliable and highly accurate results.

The silicone impression for standardizing the position of the tooth versus the long cone of the X-ray machine was helpful in measurement of the dimensions for evaluation of apical lesion modifications. This bite registration index was introduced by the previous study for one or multiple visits in endodontics [26]. This manipulation was simple, easy, and fast in clinical setting and proved that it can be served for further investigations in the endodontic therapy.

The haemostatic collagen membrane used in the present study has many advantageous properties, as it is pliable, condensable, absorbable, and easy to manipulate. Either in the wet or dry situation, this material has superior tear resistance, facilitating the formation of many layers of collagen membrane that fully fill the periapical bone loss region. Although the collagen membrane has been used commonly in previous studies, this material is continuously improved for easier manipulation, multipurpose use, and affordability [7, 9, 22].

The membrane used in our study was not basically different from other membranes [7, 9]. However, this haemostatic collagen membrane is common and has a reasonable cost when compared with the others on the market. Availability and affordability are two advantages of this haemostatic collagen membrane. Up to now, there have been several studies using different haemostatic collagen membranes [7, 9, 11], mainly on the incisors, with very few cases on the premolars [7], and these are only case reports. There is one study on the premolar, using a triple antibiotic paste without collagen membranes [27]. There was only one premolar in each study on apexification using an MTA apical plug [14, 15].

With the support of the operating dental microscope, the insertion of the resorbable haemostatic collagen was performed with predictable, safe, and precise manipulation, ensuring the safety, facilitation, and agility of the following MTA placement into the apical segment of the root canal. In the present study, all MTA placement procedures were performed only once and successfully passed the following digital X-ray check step with the operator’s handling under the dental microscope.

One of the major drawbacks of the present study was the small sample size, although it was calculated in the Methods section. This limitation is common in contemporary studies because of the rareness of this condition. Another disadvantage of the present study was the confined ethical issues, resulting in the examination on X-ray using only the periapical radiograph rather than cone-beam computed tomography (CBCT). The high level of radiation exposure was the main reason for not scanning the periapical lesion by CBCT. The follow-up period was rather short, only 9 months, for the all-aspect evaluation of the procedure’s clinical outcomes and radiographic results. Further investigation with a larger sample size, longer follow-up period, and using CBCT should be performed to obtain strong evidence supporting this procedure.

## Conclusion

The combination of MTA apical plug and haemostatic collagen membrane effectively treated dens evaginatus affected teeth with apical lesions, and open apices.

## Data Availability

The datasets used and/or analyzed during the current study are available from the corresponding author on reasonable request.

## Abbreviations

MTA: Mineral trioxide aggregate
ICC: Intra-class correlation
CBCT: Cone-beam computed tomography
